# Effect of Peel Ply on Resin Flow during Vacuum Infusion

**DOI:** 10.3390/ma16124421

**Published:** 2023-06-15

**Authors:** Sehun An, Jung-soo Kim, Hyung Doh Roh, Wie-Dae Kim, Jungwan Lee, Moon-Kwang Um

**Affiliations:** 1 Composites Research Division, Korea Institute of Materials Science (KIMS), 797 Changwon-daero, Seongsan-gu, Changwon-si 51508, Gyeongsangnam-do, Republic of Korea; anse0327@gmail.com (S.A.); kjs3832@kims.re.kr (J.-s.K.); hdroh@kims.re.kr (H.D.R.); 2 Department of Aerospace Engineering, Pusan National University, 2 Busandaehak-ro 63beon-gil, Geumjeong-gu, Busan 46241, Gyeongsangnam-do, Republic of Korea; wdkim@pusan.ac.kr

**Keywords:** peel ply, permeability, process simulation, vacuum infusion

## Abstract

Although various simulations have been conducted for the vacuum infusion process, most of the studies have considered only fabrics and flow medium and ignored the effect of peel ply. However, peel ply can affect resin flow because it is placed between the fabrics and flow medium. To verify this, permeability of two types of peel plies was measured, and it was found that the permeability between the peel plies differed significantly. Moreover, the permeability of the peel plies was lower than that of the carbon fabric; thus, peel ply can cause a bottleneck in the flow in the out-of-plane direction. Some 3D flow simulations were conducted in cases of no peel ply and for two types of the peel plies to confirm the effect of peel ply, and experiments were also conducted for two types of the peel plies. It was observed that filling time and flow pattern were highly dependent on the peel plies. The smaller permeability of peel ply has, the greater effect of peel ply is. These results indicate that the permeability of peel ply is one of the dominant factors and should be considered in process design in vacuum infusion. Additionally, by adding one layer of peel ply and applying permeability, the accuracy of flow simulations can be improved for filling time and pattern.

## 1. Introduction

The autoclave process is traditionally used for producing carbon fiber-reinforced plastic (CFRP), but it has high production costs [1]. Less-expensive processes that do not require an autoclave have been developed, such as the vacuum infusion process (VIP). The VIP cures composites after impregnating liquid resin into dry carbon fabric under vacuum pressure [2,3]. Since the carbon fabric exhibits different permeabilities depending on material characteristics, such as a weave pattern and the presence of a binder, a feasibility check of impregnation is needed before product manufacturing.

Flow simulation is an efficient method used to predict resin flow and filling time during the VIP. Flow simulation reduces costs and time-consuming work, such as a process design based on trial-and-error, and helps to optimize the locations and numbers of the inlets and vents [4]. In flow simulations, the permeability of materials is a key factor in the determination of the filling time [5]; therefore, it is important to acquire accurate permeability of the materials. Endruweit and Advani et al. attempted to reduce the deviation in permeability values by conducting standardized tests with the same specimens and molds of the same shape [6,7,8].

In the VIP, a flow medium, which is a highly permeable porous material, is used to quickly supply resin to the fabric, and a peel ply is placed between the carbon fabric and flow medium to prevent product damage during demolding [9]. However, most studies have considered only the permeability of the carbon fabric and flow medium, although peel ply is an essential material in VIP. This is because peel ply is very thin (<<0.1 mm) compared to the flow medium and fabric. Moreover, the fabrics account for most of the volume, and the flow medium has the highest permeability. Therefore, in most of the reported studies for verifying the accuracy of flow simulation [10,11,12,13] or those on numerical modeling of multilayer preform [14,15], peel ply was not considered.

Few previous studies have considered the effect of peel ply. Poodts et al. [16] investigated the effect of the peel ply used in the VIP, but they assumed three-dimensional (3D) stacked materials, which comprised fabrics, peel ply, and flow medium, as an equivalent one material with two-dimensional (2D) flow. Apparent in-plane permeability was measured and used for simulations. Joemon et al. [17] tried to find an efficient method of numerical modeling depending on compaction pressures by observing and measuring the in-plane flow of the preform including peel ply. Although the studies considered peel ply, all materials in the VIP were considered as one material and assumed as 2D flow. However, considering that the resin infused into the flow medium fills the carbon fabric by passing through the peel ply, the effect of the peel ply on the flow should be analyzed from a 3D flow point of view; therefore, the permeability of the peel ply should be directly measured (i.e., *k*_1,2,3_ should be quantified).

**Figure 1:**
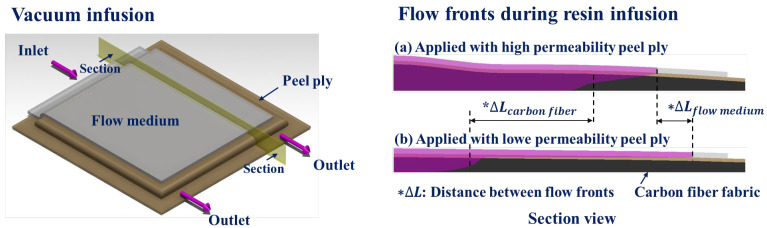
Differences of flow fronts depending on permeability of peel ply in the VIP2.

In this study, flow simulations and experiments were conducted for two types of peel plies as shown in Figure 1. To quantify the effect of the peel ply on resin flow in VIP, the permeability of two types of commercial peel plies was measured, and the cause of the difference in permeability was analyzed. Using the permeability of the peel plies, carbon fabric, and flow medium, 3D flow simulations were carried out for three models assuming that (1) no peel ply, (2) peel ply with high permeability, or (3) peel ply with low permeability was applied. The simulation results were analyzed in terms of the effects of the peel ply on 3D resin flow and were verified by performing experiments with the same geometry and conditions as those used in the simulations. In addition, by measuring the pressure and thickness change inside the vacuum film during the experiment, the change in fiber volume fraction ($V_{f}$) over time was calculated and analyzed with respect to the permeability of the peel ply.

## 2. Material Characterization

The flow medium, peel ply, and carbon fabric used in the VIP are assumed to be porous media. Fluid flow in porous media follows Darcy’s law:

(1) $$\bar{q}=-\frac{\overset{=}{k}}{\mu }\;\nabla P\;\mathrm{where}\;\bar{v}=\frac{\bar{q}}{\epsilon }$$

where $\bar{q}$,$\;\bar{v}$, ϵ, μ, ∇P, and $\overset{=}{k}$ are the Darcy velocity, fluid velocity, porosity, viscosity, pressure gradient, and permeability tensor of the porous medium. In this study, the permeability and porosity of the carbon fabric and two types of peel ply were measured, and the flow characteristics of each material were analyzed. Microstructural analysis of the two peel plies was also performed to understand their permeability differences.

### 2.1. Materials

In this study, carbon fabric was used as a reinforcement. The fabric had a 5H satin weave, thickness of 0.45 mm under no pressure, and areal weight of 384.5 g/m^2^. Two types of peel plies, Release Ease 234TFP and Release Ease 234TFP-1 (Airtech, Huntington Beach, CA, USA), were used, with thicknesses of 0.06 mm and 0.04 mm, respectively. Resinflow 90HT (Airtech) was used as the flow medium. KF-96 silicone oil (Shinetsu, Tokyo, Japan) was used for the permeability and VIP experiments to minimize the experimental variables. Unlike resin, silicone oil is chemically stable and incompressible and maintains a constant volume under pressure.

### 2.2. Measurement of Porosity

#### 2.2.1. Porosity Measurement of Carbon Fabric by Compressive Test

Because carbon fabric is compressible, its thickness decreases when a load is applied. As a result, the size of the internal pores decreases; the space through which the fluid can flow is narrowed, and the permeability also decreases. In VIP, because the inside of the film is under vacuum and the outside of the film is at atmospheric pressure, 1 atm of pressure is applied across the carbon fabric. $V_{f}$ is necessary in the determination of permeability and is calculated using Equation (2) from the thickness t of the laminated carbon fabric at 1 atm measured by a compressive test. Details of the compressive test were describe in Supplementary Materials Figure S1.

(2) $$V_{f}=\frac{W_{f}\times n}{\rho_{f}\times t}$$

Here, $W_{f}$ is the areal weight (384.5 g/m^2^) of one ply of the carbon fabric; *n* is the number of plies of laminated carbon fabrics (12 in this study); $\rho_{f}$ is the density of carbon fiber (1.78 g/cm^3^), and t was 4.67 mm. Measured fiber volume fraction was 55.5% under 1 atm.

#### 2.2.2. Porosity Measurement of Peel Ply by Thermogravimetric Analysis

The peel plies consisted of woven glass-fibers coated with polytetrafluoroethylene (PTFE). Thus, to analyze the porosity of the peel ply, it is necessary to know the relative fractions of the glass-fibers and PTFE. Glass fiber generally has a very high melting point of approximately 840 to 1000 °C, while PTFE decomposes at a much lower temperature. Therefore, it is possible to obtain the fractions of glass and PFTE in the peel ply by comparing the weights before and after the thermal decomposition of PTFE with thermogravimetric analysis (TGA). The temperature at which the decomposition of PTFE occurred was identified by a dynamic scan, for which the peel ply sample was heated from 20 to 600 °C at constant heating rate of 5 °C/min. It was observed that PTFE was fully decomposed after 530 °C. Dynamic scan results of the peel plies were described in Supplementary Materials Figure S2. To accurately measure the weight ratio of the glass fiber and PTFE in the peel ply, the moisture was removed by heating at 100 °C for 30 min, followed by heating at 530 °C for 120 min to remove the PTFE. The peel ply samples had dimensions of 5 mm × 5 mm. From the measured weight of the glass fiber $W_{gf}$ and PTFE, $W_{PTFE}$, the porosity of the peel ply, was calculated using Equation (4).

(3) $$W_{gf}=W_{total}-W_{PTFE}$$

(4) $$Porosity\;\left(\%\right)=\left(1-\frac{V_{gf}+V_{PTFE}}{V_{total}}\right)\times 100$$

### 2.3. Measurement of Permeability

**Figure 2:**
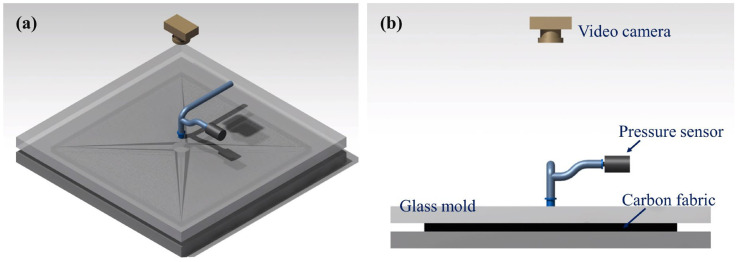
Configuration of the test setup used to measure in-plane permeability: (**a**) isometric view and (**b**) side view.

The permeability in the in-plane direction was obtained using the unsaturated flow method proposed by Chan et al. [18]. By injecting fluid through a hole in a stacked specimen, the flow fronts were monitored over time by the video camera in Figure 2. The flow distances of the main directions from the observed flow fronts were measured, and the in-plane permeability was calculated by considering the viscosity of the fluid.

**Figure 3:**
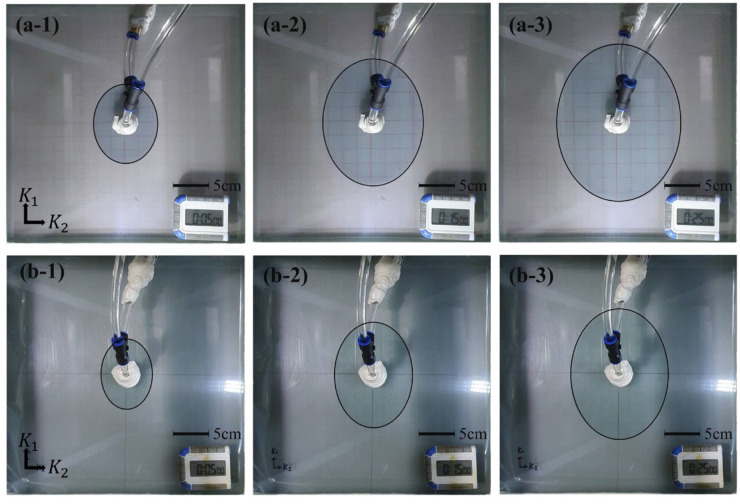
Flow front monitoring of the peel plies at various times after the start of fluid injection. Release Ease 234TFP: (**a-1**) 5 min, (**a-2**) 15 min, and (**a-3**) 25 min. Release Ease 234TFP-1: (**b-1**) 5 min, (**b-2**) 15 min, and (**b-3**) 25 min.

**Figure 4:**
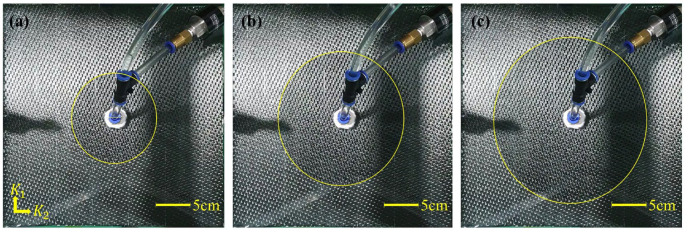
Flow front monitoring of carbon fabric over time: (**a**) 30 s, (**b**) 90 s, and (**c**) 150 s after the start of fluid injection.

To measure permeability, the test materials were cut into 300 mm × 300 mm sheets with a hole at the center. As shown in Figure 2, the specimen was placed between upper and lower rigid molds, and the thickness of the specimen was adjusted using a displacement controller. The fluid injection port was connected to the center of the upper glass mold, and the fluid was injected. During injection, a video camera located above the glass mold recorded the movement of the flow fronts over the test period. Figure 3 and Figure 4 show photographs of the flow fronts in the peel ply and carbon fabric at specific times.

**Figure 5:**
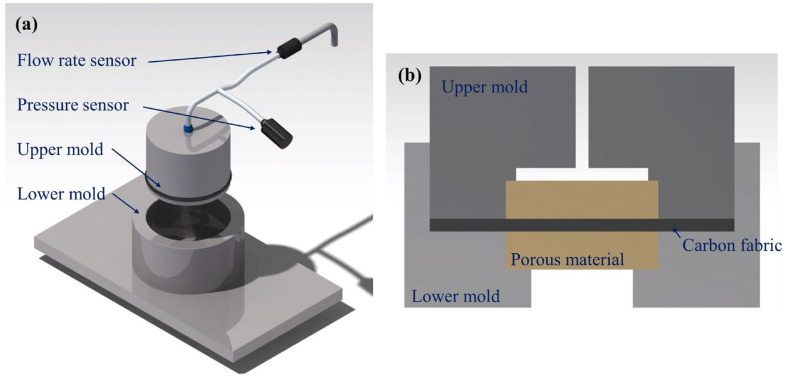
Configuration of the test setup to measure out-of-plane permeability: (**a**) isometric view and (**b**) side view.

To measure the out-of-plane permeability, the method using saturated flow suggested by Endruweit et al. was used [19]. The out-of-plane permeability test setup is shown in Figure 5. Specimens were cut into circles, and they were placed between the upper and lower molds. $V_{f}$ was controlled by adjusting the displacement of the upper mold. As shown in Figure 5b, porous metal blocks were placed at the top and bottom of the stacked specimen inside the mold to remove the flow of the boundary layer and make the velocity distribution more uniform. The permeability of the porous metal block was small enough compared with the fabric and peel ply. Pressure and flow sensors were located on the injection hose to measure the pressure and flow rate. When the fabric became wet and the flow rate became constant, the flow rate and injection pressure were measured.

### 2.4. Microstructure Observation

The Release Ease 234TFP and 234TFP-1 peel ply contain PTFE-coated woven glass fibers. Although these peel plies consist of the same materials, their microstructures depend on the weave type and PTFE content. Because the porosity characteristics predominantly determine the permeability of the peel ply, optical microscopy was used to evaluate the peel ply microstructures.

## 3. Flow Simulation of the Vacuum Infusion Process

For the flow simulation, PAM-Composites Visual-RTM 17.0 (ESI Group) software was used, which is a flow analysis program based on Darcy’s law. To analyze the effect of the peel ply on the VIP, three different models were developed using: (1) only carbon fabric and flow medium; (2) fabric, flow medium, and Release Ease 234TFP peel ply; and (3) fabric, flow medium, and Release Ease 234TFP-1 peel ply.

**Figure 6:**
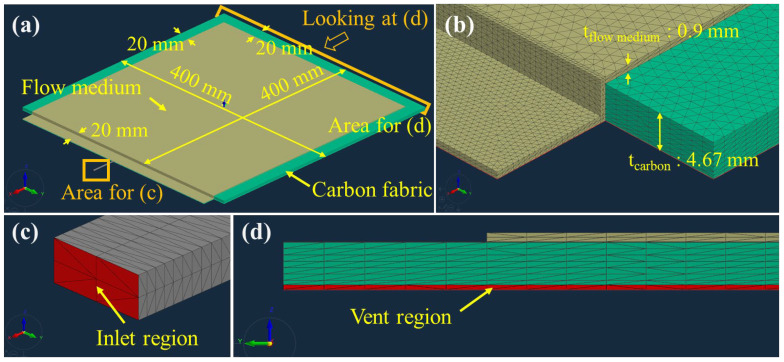
Flow simulation model without peel ply: (**a**) overall model, (**b**) 3D mesh, (**c**) inlet region, and (**d**) vent region.

Figure 6a shows the overall 3D model without peel ply. The carbon fabric was placed on the bottom with the flow medium laminated on top of the fabric. As shown in Figure 6b, a tetrahedron-type mesh was used. The mesh elements were smaller in the area close to the inlet to increase the simulation accuracy in the initial time with a high flow speed. Figure 6c,d show magnified views of the inlet and vent regions. An inlet pressure of 0.9 bar was applied. The vent region was placed on one edge of the carbon fabric, on the opposite side to the inlet, and 0 bar pressure was applied to the vent region.

**Figure 7:**
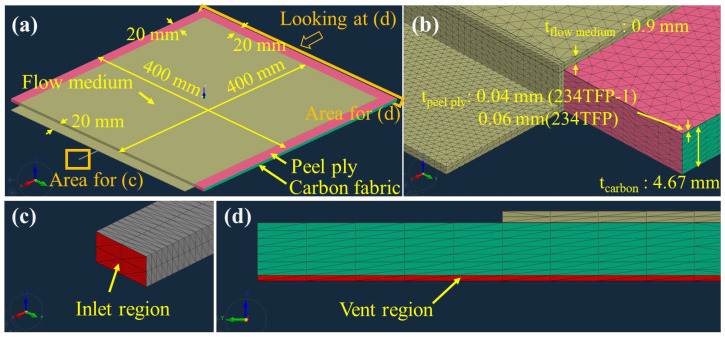
Flow simulation model with peel ply applied: (**a**) overall model, (**b**) 3D mesh, (**c**) inlet region, and (**d**) vent region.

Figure 7a shows the overall 3D model in the case of using peel ply. Unlike the model shown in Figure 6, the peel ply was added between the carbon fabric and flow medium. The boundary conditions and mesh size and shape were the same for both model types. As shown in Figure 6 and Figure 7, the thicknesses of the carbon fabric and flow medium were modeled as 4.67 mm (12 ply) and 0.9 mm, respectively, and the thicknesses of the Release Ease 234TFP and 234TFP-1 peel plies were 0.06 and 0.04 mm, shown in Figure 7b.

The permeability and porosity of the carbon fabric and peel plies used for flow simulations were measured in this study (see Table 1 and Table 2), and published values for the permeability and porosity of the flow medium were used [20]. The viscosity of the fluid was set to 0.352 Pa∙s, corresponding to that of the Shinetsu KF-96 Silicone Oil 350CS measured by a parallel plate type rheometer.

## 4. Experimental Verification of the Vacuum Infusion Process

**Figure 8:**
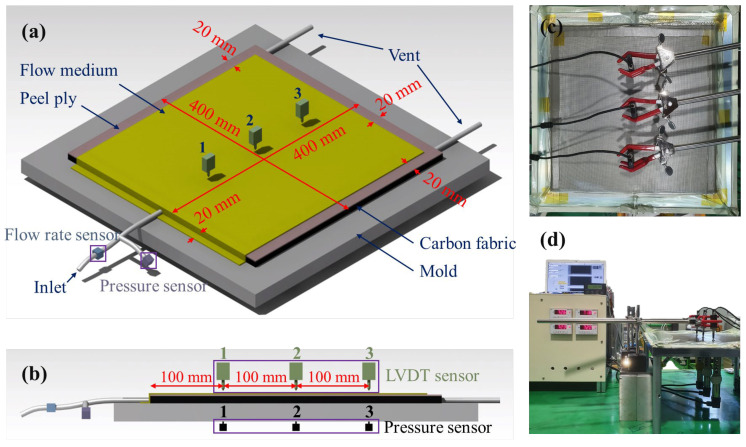
Configuration of vacuum infusion test: (**a**) isometric view, (**b**) side view, and (**c**,**d**) experimental setup.

To verify the results of the flow simulation, VIP experiments were performed under the same conditions, as shown in Figure 8. The size of the laminated carbon fabric was 400 mm × 400 mm, and the size of peel ply was slightly larger than that of the fabric to completely cover the fabric. The laminated area of the flow medium (360 mm × 400 mm) was smaller than that of the carbon fabric to minimize the fluid flow along the edge of the fabric sheet and to stabilize the flow until the end of fluid infusion. Vents were located at the farthest distance from the inlet. Two experimental cases were investigated to compare the performance of the two different peel plies. The number of stacked carbon fabric sheets was the same as that in the flow simulation models.

In the VIP, the pressure inside a vacuum film increases as resin is impregnated. It results in an increase in the thickness of the laminated fabric because the vacuum film is flexible. The pressure distribution depended on the flow characteristics, which can be affected by the type of peel ply. Thus, to verify the effect of peel ply on the thickness of the laminated fabric, the pressure and thickness were measured over time at three locations during fluid infusion. Three pressure sensors and three linear variable differential transformer (LVDT) sensors were installed in a straight line at 100 mm intervals from the inlet to record the pressure and thickness changes over time. Furthermore, after closing the vent, the pressure and changes in thickness over time were measured with the inlet open.

## 5. Results and Discussion

### 5.1. Characteristics of Carbon Fabric and Peel Plies

Table 1 lists the permeability of the materials used in the VIP. The permeabilities of both peel plies were lower than that of the carbon fabric in all directions. The in-plane permeabilities of Release Ease 234TFP and 234TFP-1 are approximately 0.20–0.28 and 0.076–0.12 times that of the carbon fabric, respectively. Furthermore, the in-plane permeability of Release Ease 234TFP is approximately 2.4–2.6 times that of Release Ease 234TFP-1. The out-of-plane permeabilities ($k_{3}$) of Release Ease 234TFP and 234TFP-1 are approximately 0.1 and 0.001 times that of the carbon fabric, respectively, showing a similar tendency to that of the in-plane permeability. However, the $k_{3}$ of Release Ease 234TFP-1 is extremely low and significantly lower than the other measured permeabilities of the peel plies. The $k_{3}$ of Release Ease 234TFP is approximately 81 times higher than that of Release Ease 234TFP-1.

The VIP maximizes the impregnated area by supplying resin to a large area in the in-plane direction via the flow medium and then filling the resin into the fabric in the out-of-plane direction, which is a very short distance compared to the in-plane dimensions. Because the resin supplied by the flow medium must flow through the peel ply to reach the carbon fabric, if the $k_{3}$ of the peel ply is lower than that of the carbon fabric, the filling rate of the resin decreases owing to a bottleneck effect. The much lower $k_{3}$ of Release Ease 234TFP-1 compared to that of the carbon fabric means that this peel ply will significantly affect the resin filling rate. Therefore, because of the very low permeability of Release Ease 234TFP-1, it takes a longer time to completely fill the resin in the carbon fabric compared to Release Ease 234TFP when other conditions are the same, such as the part dimensions and shape and flow medium dimension.

Table 2 shows the results of the TGA isothermal scan comparing the weights of the peel ply before and after PTFE was completely decomposed. From these data, the weight ratio of glass fiber and PTFE in the peel ply was calculated, and the volume fraction of the glass fiber and PTFE was obtained from the weight ratio and density of each material [21]. The remaining weight of Release Ease 234TFP after the complete decomposition of PTFE is higher than that of Release Ease 234TFP-1, consistent with the greater thickness of the former (0.06 mm) compared to the latter (0.04 mm). The measured porosities of Release Ease 234TFP and 234TFP-1 were 44.54% and 34.06%, respectively. The lower porosity of Release Ease 234TFP-1 is consistent with its lower permeability, because decreasing the porosity increases the flow resistance as the flow paths become shorter.

**Figure 9:**
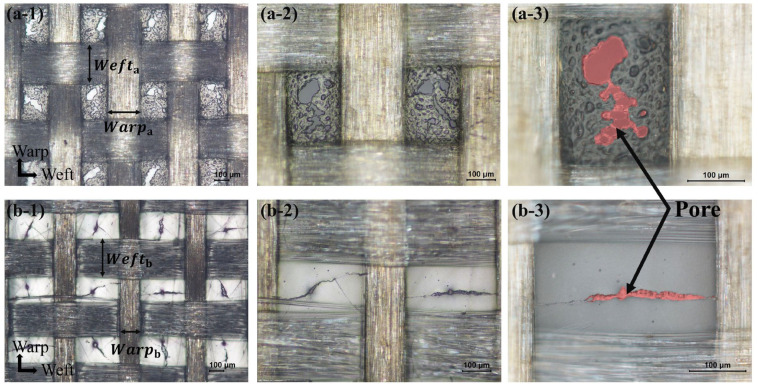
Optical microscopy images of Release Ease 234TFP: (**a-1**) 50× image, (**a-2**) 100× image, and (**a-3**) 200× image, and Release Ease 234TFP-1: (**b-1**) 50× image, (**b-2**) 100× image, and (**b-3**) 200× image.

Optical microscopy images of the two peel plies are shown in Figure 9. As shown in the 50× magnification images in Figure 9(a-1,b-1), the plain-woven glass fiber was coated with PTFE, and the empty spaces between the rows were mostly filled with PTFE. The width of the glass fiber in the weft direction was similar for both peel plies (*Weft*_a_ ≈ *Weft*_b_), but the warp width of Release Ease 234TFP was much wider than that of Release Ease 234TFP-1 (*Warp*_a_ > *Warp*_b_). These differences in the weave patterns of the glass fiber contribute to the observed differences in thickness, $V_{f}$, and porosity of the two types of peel plies. Furthermore, as shown in Table 2, Release Ease 234TFP-1 had a higher content of PTFE but lower content of glass fibers compared to Release Ease 234TFP. This implies that the spaces between the woven glass fibers in Release Ease 234TFP-1 are more completely filled with PTFE than those in the other peel ply. As shown in the 100× images (Figure 9(a-2,b-2)) and 200× images (Figure 9(a-3,b-3)), the two peel plies have different pore sizes, and the pore size of Release Ease 234TFP-1 is significantly smaller than that of the other peel ply. The porosity of Release Ease 234TFP-1 calculated from the TGA results was ~10% lower than that of Release Ease 234TFP, and the tendency observed from the microstructure is similar.

In summary, the permeability and porosity of Release Ease 234TFP-1 is lower than that of Release Ease 234TFP. The measured results are consistent with scientifically proven facts. In particular, the $k_{3}$ of Release Ease 234TFP-1 is 100 times lower than that of Release Ease234TFP due to significant filling of the spaces between the woven glass fibers with PTFE, which blocks the flow.

### 5.2. Flow Simulation and Experimental Verification

**Figure 10:**
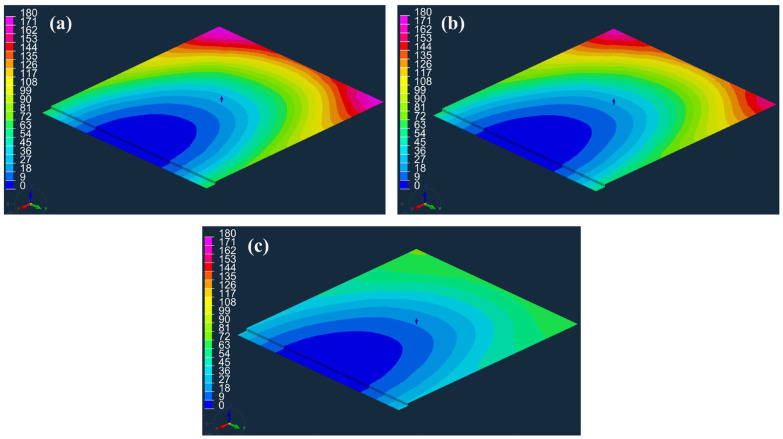
Flow fronts in the flow medium over time (s): (**a**) no peel ply applied, (**b**) Release Ease 234TFP applied, and (**c**) Release Ease 234TFP-1 applied.

Figure 10a–c show the simulated flow fronts in the flow medium over time for the cases in which no peel ply is applied, Release Ease 234TFP is applied, and Release Ease 234TFP-1 is applied, where the times required to fill the flow medium with resin were 170, 167, and 76 s. In Figure 10a,b for the cases of no peel ply and peel ply with high $k_{3}$, respectively, the fluid can easily flow in the out-of-plane direction from the flow medium to the carbon fabric. However, in Figure 10c, for the peel ply with low $k_{3}$, the peel ply interferes with the out-of-plane flow to the carbon fabric and causes the fluid to flow rapidly in the in-plane direction. Thus, it takes a shorter time to fill the flow medium with resin when Release Ease 234TFP-1, with low $k_{3}$, is used.

**Figure 11:**
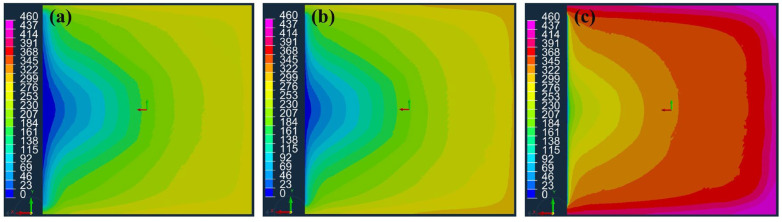
Flow fronts at the bottom of carbon fabric over time (s): (**a**) no peel ply applied, (**b**) Release Ease 234TFP applied, and (**c**) Release Ease 234TFP-1 applied.

Figure 11 shows the simulated flow fronts over time at the bottom of the carbon fabric. Figure 11a–c show the cases when no peel ply is applied, Release Ease 234TFP is applied, and Release Ease 234TFP-1 is applied, giving filling times of the carbon fabric of 295, 312, and 458 s. As shown in Table 3, the high-permeability peel ply (Release Ease 234TFP) only increased the filling time by ~6% compared to the case with no peel ply, while the low-permeability peel ply (Release Ease 234TFP-1) increased this time by ~55%. In the case of high-permeability peel ply, its influence on the process is small, and, thus, a simulation without the peel ply may be able to accurately estimate the actual process. However, when a peel ply with low permeability is used, if the peel ply is not considered, a very large error can occur in the prediction of the filling time, and incorrect flow patterns can be predicted.

**Figure 12:**
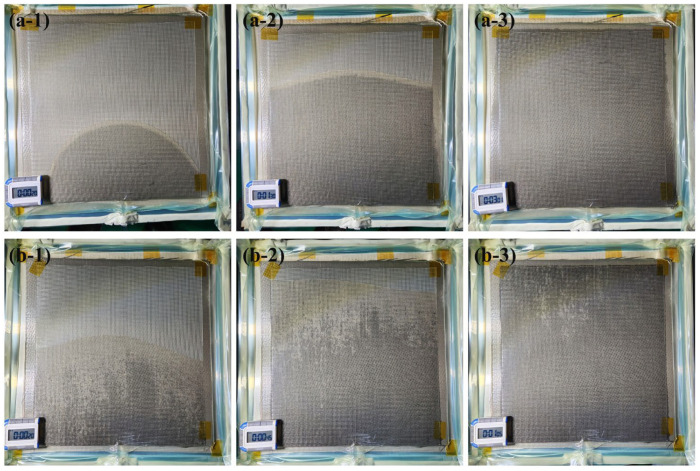
Flow fronts viewed from the top over time after resin infusion was started, as obtained from verification tests using Release Ease 234TFP: (**a-1**) 20 s, (**a-2**) 90 s, and (**a-3**) 181 s or Release Ease 234TFP-1: (**b-1**) 20 s, (**b-2**) 45 s, and (**b-3**) 65 s.

Figure 12 shows the flow fronts in the flow medium over time obtained from the experimental verification tests using the two types of peel ply. Figure 12(a-3,b-3) show the time at which the flow medium was completely filled, which took 181 and 65 s for Release Ease 234TFP and 234TFP-1, respectively. As for the simulation results, when high-permeability Release Ease 234TFP was used, it took longer for the flow medium to be completely filled compared with using low-permeability Release Ease 234TFP-1. The experimental flow fronts in the flow medium over time have similar shapes to the simulated flow fronts shown in Figure 10b,c. The filling times of the carbon fabric obtained in the experiments were 337 and 537 s when Release Ease 234TFP and 234TFP-1 were used, respectively, which is attributed to the higher $k_{3}$ of Release Ease 234TFP.

Comparing the filling time (295 s) obtained by the simulation without peel ply with the experimental filling times with the peel plies (337 s for Release Ease 234TFP and 537 s Release Ease 234TFP-1), the simulation result showed differences of 12% and 45%, respectively. As expected from the simulation results, the difference was acceptably small between the simulation without peel ply and the experiment with Release Ease 234TFP. However, when Release Ease 234TFP-1 was used, the filling time could not be accurately predicted by the model. In contrast, when the peel ply was considered in the flow simulation, the filling-time differences between the simulation and experiment for Releases Ease 234TFP and 234TFP-1 were quite low, approximately 7% and 14%, respectively. Therefore, to improve the predictive accuracy of VIP flow simulation models, peel ply should be considered, especially for low-permeability peel plies.

During the VIP, the resin is supplied over a wide area in the in-plane direction via the flow medium, which has a very high permeability to maximize the filling area, and then filling of the carbon fabric with the resin subsequently occurs in the out-of-plane direction. Therefore, if the flow medium is considered as a fluid-supply element, we can assume a 1D-series linear flow in the out-of-plane direction of the peel ply and carbon fabric. In a series linear flow, the average permeability can be expressed as the sum of the lengths and permeability values of each element, as shown in Equation (5) [22]. An example of derivation of below equation was described in Supplementary Materials Figure S3.

(5) $$k_{3,avg}=\frac{\sum L_{i}}{\sum \frac{L_{i}}{k_{i}}}$$

Here, $L_{i}$ and $k_{i}$ are the length and permeability of the element, respectively. If the peel ply and carbon fabric are assumed to have a series connection in the out-of-plane direction, $k_{3,avg}$ can be represented as shown in Equation (6).

(6) $$k_{3,avg}=\frac{\;L_{c}+L_{p}}{\frac{L_{c}}{k_{c}}+\frac{L_{p}}{k_{p}}}$$

where L and k represent the thickness and out-of-plane permeability, respectively, and the subscripts *c* and *p* refer to carbon fabric and peel ply, respectively.

**Figure 13:**
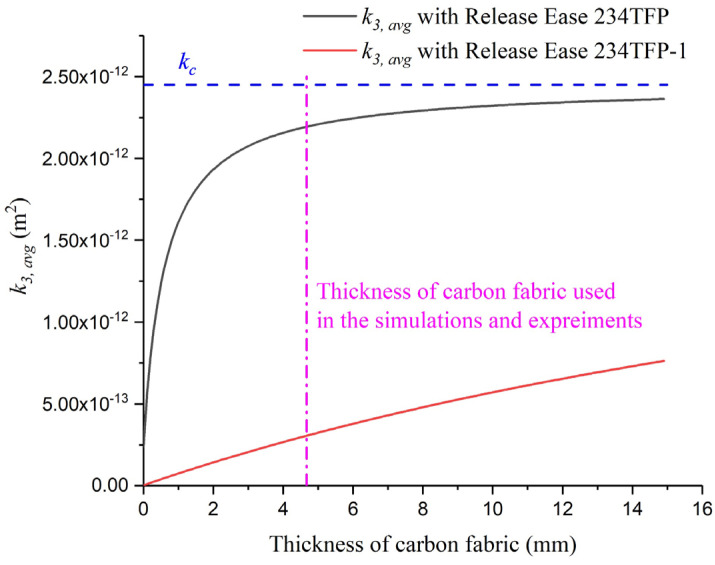
Average out-of-plane permeability as a function of thickness of the carbon fabric obtained using the two different peel piles.

Figure 13 shows the calculated $k_{3,avg}$ as a function of $L_{c}$ for the two different peel plies. The blue dashed line corresponds to $k_{c}$ and the green dashed line corresponds to the $L_{c}$ used in the simulations and experiments (4.67 mm). When Release Ease 234TFP was applied, $k_{3,avg}$ gradually converged to $k_{c}$ as $L_{c}$ increased, whereas $k_{3,avg}$ increased linearly when Release Ease 234TFP-1 was applied. When $k_{p}$ is low enough to ignore $L_{c}$ and $L_{p}$, Equation (6) can be written as Equation (7). When $L_{p}$ is very thin compared with $L_{c}$, Equation (7) can be written as Equation (8).

(7) $$\mathrm{If}\;\frac{L_{p}}{k_{p}}\;\gg \;\frac{L_{c}}{k_{c}}\;\to \;k_{3,avg}\;≅\;k_{p}\frac{L_{p}+L_{c}}{L_{p}}$$

(8) $$\mathrm{If}\;L_{c}\;\gg \;L_{p}\;\to \;k_{3,avg}\;≅\;k_{p}\frac{L_{c}}{L_{p}}$$

In the case of using Release Ease 234TFP-1 and the carbon fabric, because of $\frac{L_{p}}{k_{p}}\;\gg \;\frac{L_{c}}{k_{c}}$ (1.3 × 10^10^ ≫ 1.9 × 10^9^ m^−1^) and $L_{c}\gg$ $L_{p}$ (4.67 ≫ 0.04 mm), $k_{3,avg}$ increases with increasing $L_{c}$.

At 4.67 mm thickness, the difference between $k_{c}$ and $k_{3,avg}$ with Release Ease 234TFP was approximately 10%, and the permeability values were similar. This is consistent with the small difference in filling times (~6%) obtained by the flow simulation without peel ply and with Release Ease 234TFP applied. Conversely, it can be seen that $k_{3,avg}$ with Release Ease 234TFP-1 has a very low value of about 0.12 times compared to the $k_{3}$ of carbon fabric. Because of this, when Release Ease 234TFP-1 is applied in the VIP, the filling time is greatly increased. Thus, by calculating $k_{3,avg}$ at a target thickness of carbon fabric and comparing it with $k_{c}$, we can determine whether a 3D model of peel ply is needed before flow simulations.

### 5.3. Variations in the Thickness of Carbon Fabric during Resin Infusion

**Figure 14:**
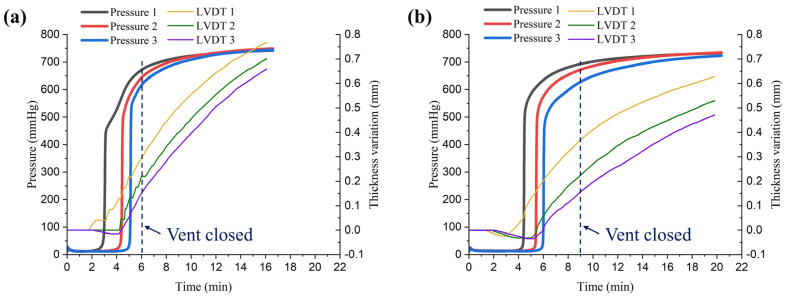
Pressure and thickness of the carbon fabric during infusion experiments using different peel plies: (**a**) Release Ease 234TFP or (**b**) Release Ease 234TFP-1.

In the VIP using a flexible vacuum film, the carbon fabric can swell due to a change of internal pressure by the infused resin. To lower the risk of voids being formed in the product, the inlet was left open for a certain amount of time after the vents were closed to increase the inner pressure and allow all areas to converge to the inlet pressure as the hydrostatic state [23,24,25]. Therefore, to measure the pressure and thickness changes during infusion and after closing the vents, experiments were conducted with the two types of peel plies, as shown in Figure 8. The measured pressure and thickness over time are shown in Figure 14. The pressure and thickness increased as the resin was infused and continued to increase even after the vents were closed. When Release Ease 234TFP was used, the vent was closed after 6 min, and for Release Ease 234TFP-1, the vent was closed after 9 min. When using Release Ease 234TFP, the pressure converged more quickly after closing the vent, and the thickness increased faster compared to the case of Release Ease 234TFP-1. This was due to the high permeability of the 234TFP peel ply easily transferring the inlet pressure to low-pressure areas of the carbon fabric.

In Table 4, $V_{f}$ values calculated from the thickness measured by the LVDT sensors are shown over time after closing the vent. Immediately after closing the vent, the average $V_{f}$ in case of using Release Ease 234TFP was 0.29% higher than that when Release Ease 234TFP-1 was used. However, 5 min after closing the vent, the average $V_{f}$ for Release Ease 234TFP-1 was ~1% higher than that of the other peel ply, and the difference increased to 1.67% 10 min after closing the vent. These results show that using a lower-permeability peel ply can increase the filling time of the VIP, but it can also be an effective method to minimize the reduction of $V_{f}$.

## 6. Conclusions

Although most of studies have ignored the influence of peel ply when simulating the flow of the VIP, this study proved that the permeability of the peel ply has a significant effect on the resin flow during the VIP through flow simulations and experiments. The measured permeability of two commercial peel plies were both lower than that of the carbon fabric, and, thus, the peel ply interfered with the resin flow from the flow medium to the fabric. In particular, the $k_{3}$ of Release Ease 234TFP-1 was approximately 0.001 times lower than that of carbon fabric and 0.1 times lower than that of Release Ease 234TFP.

The flow simulation without peel ply estimated a filling time close to that obtained with the high-permeability peel ply (~6% difference) but greatly underestimated the filling time for the system using the low-permeability peel ply (~35% difference). Moreover, flow patterns were different; the flow front in the flow medium was faster, but filling time was longer when the high-permeability peel ply was applied. Therefore, to predict the flow patterns and filling time accurately, a 3D model of the peel ply and the permeability of the peel ply should be included in the simulation model, especially for low-permeability peel plies. The experimental results confirmed this trend. Additionally, experimental and simulation results were observed to be similar for both peel plies.

Further, the average permeability of the peel ply and fabrics, which was calculated by assuming a 1D series linear flow in the out-of-plane direction, can be used to determine whether the peel ply should be reflected in flow simulations by comparing with the out-of-plane permeability of the carbon fabric. In addition, it was found that the permeability of the peel ply also affects the fiber volume fraction. After the vents were closed during resin infusion, the peel plies with lower permeability showed less reduction of fiber volume fraction. Although a lower-permeability peel ply results in longer filling time, it facilitates a higher fiber volume fraction.

## Figures and Tables

**Table 1:** Measured permeabilities of test materials.

Permeability	Release Ease 234TFP	Release Ease 234TFP-1	Carbon Fabric	Flow Medium [20]
*k*_2_/*k*_1_	0.57	0.51	0.79	0.34
$k_{1}\;\left[m^{2}\right]$	2.32 × 10^−11^	9.71 × 10^−12^	8.35 × 10^−11^	2.40 × 10^−8^
	0.28 ^1^	0.12 ^1^		
	2.4 ^2^			
$k_{2}\;\left[m^{2}\right]$	1.32 × 10^−11^	5.00 × 10^−12^	6.61 × 10^−11^	8.19 × 10^−9^
	0.20 ^1^	0.076 ^1^		
	2.6 ^2^			
$k_{3}\;\left[m^{2}\right]$	2.40 × 10^−13^	2.96 × 10^−15^	2.45 × 10^−12^	1.00 × 10^−6^
	0.098 ^1^	0.0012 ^1^		
	81 ^2^			

^1^ Permeability ratio of the peel ply to the carbon fabric. ^2^ Permeability ratio of Release Ease 234TFP of Release Ease 234TFP-1.

**Table 2:** TGA measurement results under isothermal conditions and volume fractions and porosities of the glass fiber and PFTE for each peel ply. Densities of glass fiber and PTFE were assumed to be 2.48 and 2.20 g/cm^3^, respectively.

	Release Ease 234TFP			Release Ease 234TFP-1		
Specimen number	1	2	3	1	2	3
Initial weight [mg]	1.589	1.623	1.588	1.314	1.266	1.334
Weight of glass fiber [mg]	1.098	1.103	1.054	0.590	0.578	0.597
Weight of PTFE [mg]	0.491	0.520	0.534	0.724	0.688	0.737
Thickness [mm]	0.06			0.04		
Average volume fraction glass fiber [%]	36.13			27.81		
Average volume fraction PTFE [%]	19.33			38.13		
Porosity [%]	44.54			34.06		

**Table 3:** Comparison of simulation and experimental results.

Filling Time (s)		
Use of peel ply	Simulation	Experiment
Not	295	-
Release Ease 234TFP	312	337
Release Ease 234TFP-1	458	537

**Table 4:** Fiber volume fractions (%) calculated from the measured thickness during infusion with the two different peel plies.

	Release Ease 234TFP			Release Ease 234TFP-1		
	LVDT 1	LVDT 2	LVDT 3	LVDT 1	LVDT 2	LVDT 3
Vent closed	52.21	53.01	53.77	51.46	52.96	53.70
5 min after closing vent	49.19	50.08	50.64	49.92	51.12	51.92
10 min after closing vent	47.69	48.28	48.67	49.06	50.01	50.59

## Supplementary Materials

The following supporting information can be downloaded at: https://www.mdpi.com/article/10.3390/ma16124421/s1, Figure S1: Setup of compressive test; Figure S2: Thermogravimetric measurement results at constant heating rate of 5 °C/min for the two different peel piles: (a) Release Ease 234TFP and (b) Release Ease 234TFP-1; Figure S3: Similarity of average permeability and average resistance in series. References [22,26] are cited in the Supplementary Materials.
