# Supplementary material for: Effect of Peel Ply on Resin Flow during Vacuum Infusion
Source: Materials (Basel). 2023 Jun 15;16(12):4421. doi: 10.3390/ma16124421 (PMC10303705; doi:10.3390/ma16124421)
Supplement: Supplementary file 1 [file materials-16-04421-s001.zip › materials-2402793-supplementary.pdf]

materials

MDPI

Supporting Information

Effect of Peel Ply on Resin Flow during Vacuum Infusion

Sehun An [1,2], Jung-soo Kim [1], Hyung Doh Roh [1], Wie-Dae Kim [2], Jungwan Lee [1,*] and Moon-Kwang Um [1,*]

[1] Composites Research Division, Korea Institute of Materials Science (KIMS), 797 Changwon-daero, Seongsan-gu, Changwon-si 51508, Gyeongsangnam-do, Republic of Korea; anse0327@gmail.com (S.A.); kjs3832@kims.re.kr (J.-s.K.); hdroh@kims.re.kr (H.D.R.)

[2] Department of Aerospace Engineering, Pusan National University, 2 Busandaehak-ro 63beon-gil, Geumjeong-gu, Busan 46241, Gyeongsangnam-do, Republic of Korea; wdkim@pusan.ac.kr

* Correspondence: jwlee0626@kims.re.kr (J.L.); umk1693@kims.re.kr (M.-K.U.)

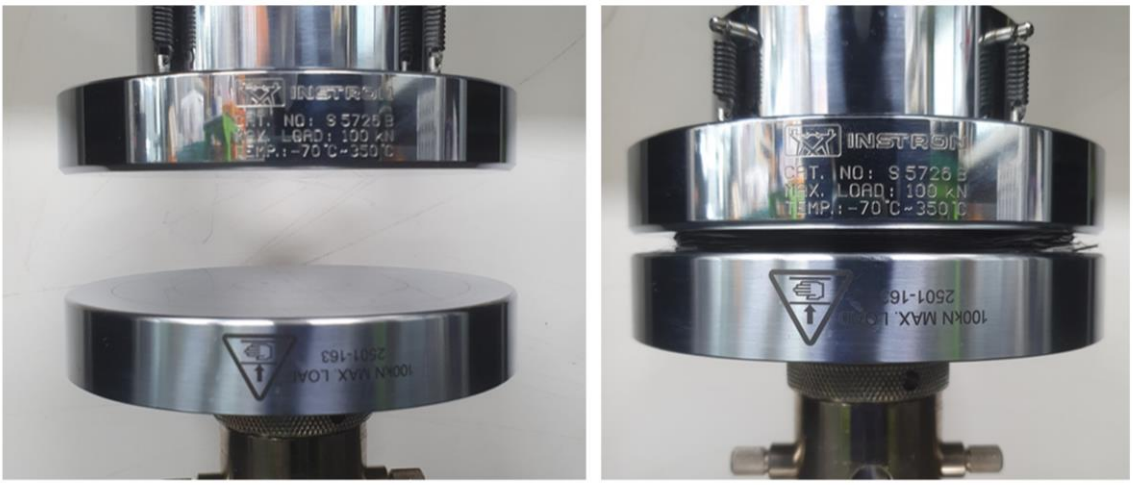

Figure S1. Setup of compressive test.

In Section 2.2.1, a fiber volume fraction was calculated by compressive tests. To conduct the compressive test, carbon fabrics were cut with 100 mm by 100 mm and 12 plies of the carbon fabrics were laminated. Displacement was set to zero when two compression plates were in contact and a positive value when the compression plates were apart. Carbon fabrics in a dry state were pressed using a universal testing machine until normal pressure reached 1 atm. At 1 atm, a thickness of the carbon fabrics was obtained from measured displacement and the thickness was used to analyze fiber volume fraction.

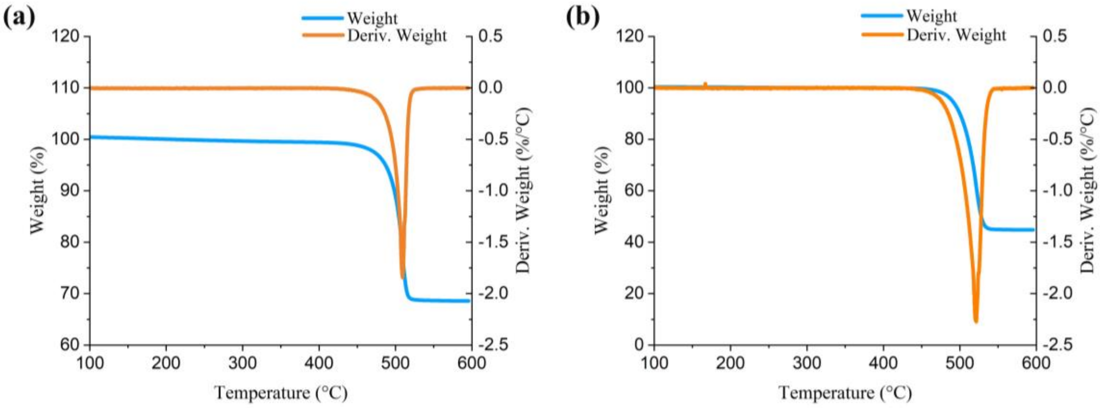

Figure S2. Thermogravimetric measurement results at constant heating rate of 5 ℃/min for the two different peel piles: (a) Release Ease 234TFP and (b) Release Ease 234TFP-1.

As mentioned Section 2.2.2, the thermal decomposition point of the glass-fiber fabric and PTFE, which constitute the peel plies, are different. To determine complete decomposition temperature of PTFE, thermogravimetric measurements were carried out for the two different peel piles at constant heating rate of 5 °C/min from 20 to 600 °C. As shown in Figure S2, a rapid weight change occurred above 450 °C and weight converged to a specific value above 530 °C for both two type of peel plies. Thus, 530 °C was used as isothermal conditions of experiments to completely remove PTFE.

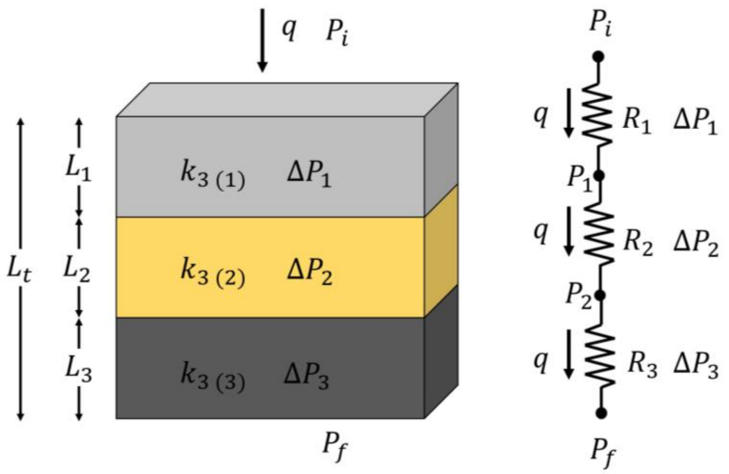

Figure S3. Similarity of average permeability and average resistance in series.

Figure S3 shows average permeability (k_3) of flow units in series [1]. This corresponds to the series linear Flow-Harmonic average [2], and can be approached by Ohm's law on the current flowing in series along the wire. Flow (q), Pressure (P) and resistance to flow ($\frac{L}{kA}$) can be represented as current (I), voltage (V) and resistance (R_e), respectively. The pressure difference for each material can be expressed as follows by Darcy's law.

$$\Delta P = \Delta P_1 + \Delta P_2 + \Delta P_3 \quad \text{(S1)}$$

$$\Delta P = \frac{q\mu}{A}\frac{L_t}{k_{avg}} \quad \text{(S2)}$$

Substituting Equations (S2) into Equation (S1) we can develop the following expression.

$$\frac{q\mu}{A}\frac{L}{k_{avg}} = \frac{q\mu}{A}\frac{L_1}{k_{3(1)}} + \frac{q\mu}{A}\frac{L_2}{k_{3(2)}} + \frac{q\mu}{A}\frac{L_3}{k_{3(3)}} \quad \text{(S3)}$$

References

1. Kedem, O.; Katchalsky, A. Permeability of composite membranes. Part 3.—Series array of elements. *Trans. Faraday Soc.* **1963**, *59*, 1941–53.

2. Ahmed, T. *Reservoir Engineering Handbook*; Elsevier Inc: Oxford, UK, 2019.
